# Kinetic Energy of a Free Quantum Brownian Particle

**DOI:** 10.3390/e20020123

**Published:** 2018-02-12

**Authors:** Paweł Bialas, Jerzy Łuczka

**Affiliations:** 1 Institute of Physics, University of Silesia, 41-500 Chorzów, Poland; 2 Silesian Center for Education and Interdisciplinary Research, University of Silesia, 41-500 Chorzów, Poland

**Keywords:** quantum Brownian motion, kinetic energy, equipartition theorem

## Abstract

We consider a paradigmatic model of a quantum Brownian particle coupled to a thermostat consisting of harmonic oscillators. In the framework of a generalized Langevin equation, the memory (damping) kernel is assumed to be in the form of exponentially-decaying oscillations. We discuss a quantum counterpart of the equipartition energy theorem for a free Brownian particle in a thermal equilibrium state. We conclude that the average kinetic energy of the Brownian particle is equal to thermally-averaged kinetic energy per one degree of freedom of oscillators of the environment, additionally averaged over all possible oscillators’ frequencies distributed according to some probability density in which details of the particle-environment interaction are present via the parameters of the damping kernel.

## 1. Introduction

One of the enduring milestones of classical statistical physics is the theorem of the equipartition of energy [1], which states that energy is shared equally amongst all energetically-accessible degrees of freedom of a system and relates average energy to the temperature *T* of the system. In particular, for each degree of freedom, the average kinetic energy is equal to $E_{k}=k_{B}T/2$, where $k_{B}$ is the Boltzmann constant. This relation is exploited in various aspects of many areas of physics, chemistry and biology. However, in many cases, it is applied in an unjustified way forgetting about assumptions used in proving this theorem. One can notice confusion and mess, in particular in the case of quantum systems. In the standard course of classical statistical physics, the equality $E_{k}=k_{B}T/2$ is derived under the following conditions:

1. The system is at thermal equilibrium of temperature *T*.
2. The state of the system is described by the Gibbs canonical probability distribution.
3. The Gibbs probability distribution describes an equilibrium state of the system in the limit of weak coupling with the thermostat.
4. The Gibbs probability distribution does not depend on the system-thermostat coupling constant.

It should be stressed that in classical statistical physics, the equality $E_{k}=k_{B}T/2$ is universal: it does not depend either on the number of particles of the system or on the single-particle potential $U({\vec{r}}_{i})$ in which the *i*-th particle dwells, as well as it does not depend on the form of mutual interaction $U({\vec{r}}_{i},{\vec{r}}_{j})$ between *i*-th and *j*-th particles of the system.

In quantum physics, the problem is more complicated. In the weak coupling limit, the density operator (or the density matrix) ρ for the canonical ensemble describes a thermal equilibrium state and has the form:

(1) $$\rho =\frac{1}{Z}e^{-H/k_{B}T},\;Z=\mathrm{Tr}\left(e^{-H/k_{B}T}\right),$$

where *H* is a Hamiltonian of the system. In the book by Feynman [2], one can find Expression (2.88) for average kinetic energy $E_{k}\left(\omega_{0}\right)$ of a quantum harmonic oscillator of the eigenfrequency $\omega_{0}$. It has the form:

(2) $$E_{k}\left(\omega_{0}\right)=\frac{1}{2m}\langle p^{2}\rangle =\frac{\hbar \omega_{0}}{4}\coth\frac{\hbar \omega_{0}}{2k_{B}T},$$

where *p* is momentum and *m* is the mass of the oscillator. The Hamiltonian of the oscillator has the well-known form:

(3) $$H=\frac{p^{2}}{2m}+\frac{m\omega_{0}^{2}x^{2}}{2}.$$

We can perform the limit $\omega_{0}\to 0$, which corresponds to the Hamiltonian of a free particle. In this limit, Equation (2) assumes the form:

(4) $$E_{k}=\frac{1}{2}k_{B}T.$$

It is the same expression as for the classical free particle. However, Equations (2) and (4) are different. This means that for quantum systems, in contrast to a classical case, the average kinetic energy depends on the potential U(x), even in the weak coupling limit. If the weak coupling limit does not hold, the problem is even much more complicated. It is the aim of this paper to discuss the question of equipartition energy for an arbitrary system-thermostat coupling. We study only one specific and as simple as possible model of a quantum open system to present basic concepts and ideas. Therefore, we consider a free quantum particle coupled to its environment, which is modeled as a collection of harmonic oscillators of temperature *T*. This old clichéd system-environment model [3] has been re-considered many, many times by each next generation of physicists [4]. However, it is still difficult to find a transparent presentation of this fundamental issue of the quantum statistical physics focused on the kinetic energy. To achieve the aim, we try to use the simplest techniques and methods to make the paper consistent and self-contained, and we neglect many unnecessary and redundant aspects of the theory.

The remaining part of the paper is organized as follows. In Section 2, we describe the model and derive the integro-differential Generalized Langevin Equation (GLE); in Section 3, the fluctuation-dissipation relation is presented; the form of the dissipation integral kernel of GLE is described in Section 4; in Section 5, we convert the integro-differential Langevin equation into a set of differential equations; the equipartition theorem is discussed in Section 6; some selected physical regimes are analyzed in Section 7; we conclude the work with a brief résumé in Section 8; in Appendix A, Appendix B and Appendix C, we present some auxiliary calculations.

## 2. Hamiltonian Model and Generalized Langevin Equation

A paradigmatic model of a one-dimensional quantum Brownian motion consists of a particle of mass *M* subjected to the potential U(x) and interacting with a large number of independent oscillators, which form a thermostat (environment) E of temperature *T* being in an equilibrium canonical (Gibbs) state. The quantum-mechanical Hamiltonian of such a total system can be written in the form [3,4,5]:

(5) $$H=\frac{p^{2}}{2M}+U\left(x\right)+\sum_{i}\left[\frac{p_{i}^{2}}{2m_{i}}+\frac{m_{i}\omega_{i}^{2}}{2}{\left(q_{i}-\eta_{i}x\right)}^{2}\right],$$

where the coordinate and momentum operators {x,p} refer to the Brownian particle and $\left\{q_{i},p_{i}\right\}$ are the coordinate and momentum operators of the *i*-th heat bath oscillator of mass $m_{i}$ and the eigenfrequency $\omega_{i}$. The parameter $\eta_{i}$ characterizes the interaction strength of the particle with the *i*-th oscillator. There is the counter-term, the last term proportional to $x^{2}$, which is included to cancel a harmonic contribution to the particle potential. All coordinate and momentum operators obey canonical equal-time commutation relations.

The next step is to write the Heisenberg equations of motion for all coordinate and momentum operators. For the Brownian particle, the Heisenberg equations are:

(6) $$\begin{array}{cc} \dot{x}\equiv \frac{dx}{dt} & =\frac{i}{\hbar }\left[H,x\right]=\frac{p}{M}, \end{array}$$

(7) $$\begin{array}{cc} \dot{p}\equiv \frac{dp}{dt} & =\frac{i}{\hbar }\left[H,p\right]=\frac{i}{\hbar }\left[U(x),p\right]+\sum_{i}c_{i}\left(q_{i}-\eta_{i}x\right), \end{array}$$

where:

(8) $$c_{i}=\eta_{i}m_{i}\omega_{i}^{2}.$$

For the environment operators, one gets:

(9) $$\begin{array}{cc} \dot{q_{i}} & =\frac{p_{i}}{m_{i}}, \end{array}$$

(10) $$\begin{array}{cc} \dot{p_{i}} & =-m_{i}\omega_{i}^{2}q_{i}+c_{i}x. \end{array}$$

What we need in Equation (7) is the solution $q_{i}=q_{i}\left(t\right)$, which can be obtained from Equations (9) and (10) with the result (see [6]):

(11) $$q_{i}\left(t\right)=q_{i}\left(0\right)\cos\left(\omega_{i}t\right)+\frac{p_{i}\left(0\right)}{m_{i}\omega_{i}}\sin\left(\omega_{i}t\right)+\frac{c_{i}}{m_{i}\omega_{i}}\int_{0}^{t}\sin\left[\omega_{i}\left(t-s\right)\right]x\left(s\right)ds.$$

The following step is to integrate by parts the last term in Equation (11) and insert it into Equation (7) for p=p(t). Using Equation (6), after some algebra, one can obtain an effective equation of motion for the particle coordinate x(t). It is called a generalized Langevin equation and has the form:

(12) $$\begin{array}{c} M\ddot{x}\left(t\right)+\int_{0}^{t}\gamma \left(t-s\right)\dot{x}\left(s\right)\;ds=-U^{\prime }\left(x\left(t\right)\right)-\gamma \left(t\right)x\left(0\right)+F\left(t\right), \end{array}$$

where $U^{\prime }\left(x\right)$ denotes differentiation with respect to *x*, γ(t) is a dissipation function (damping or memory kernel) and F(t) denotes the random force,

(13) $$\begin{array}{cc} \gamma (t-s) & =\sum_{i}\frac{c_{i}^{2}}{m_{i}\omega_{i}^{2}}\cos\left(\omega_{i}\left(t-s\right)\right), \end{array}$$

(14) $$\begin{array}{cc} F(t) & =\sum_{i}c_{i}\left[q_{i}\left(0\right)\cos\left(\omega_{i}t\right)+\frac{p_{i}\left(0\right)}{m_{i}\omega_{i}}\sin\left(\omega_{i}t\right)\right]. \end{array}$$

The dynamics of the quantum Brownian particle is therefore described by a stochastic integro-differential equation for the coordinate operator x(t). Probably Magalinskij [3] was the first, in 1959, to derive the integro-differential Equation (12) and formulated the problem in the above way. Next, from 1966, a series of papers were published on this topic, but a complete list of papers is too long to present here. We cite a part of them [7,8,9,10,11,12]. Generally, it is difficult to analyze this kind of integro-differential equation for operators. However, in the case of a free Brownian particle (when U(x)=0) or for a harmonic oscillator (when $U\left(x\right)\propto x^{2}$), Equation (12) can be solved exactly, at least in a formal way. In the classical case, equations like Equation (12) describe non-Markovian stochastic processes [5]. In the quantum case, there is no good definition of Markovian or non-Markovian processes, and therefore, a classification with respect to these notions is not constructive.

## 3. Fluctuation-Dissipation Theorem

In the standard approach, it is assumed that the initial state ρ(0) of the total system is uncorrelated, i.e., $\rho \left(0\right)=\rho_{S}⊗\rho_{T}$, where $\rho_{S}$ is an arbitrary state of the Brownian particle and $\rho_{T}$ is an equilibrium canonical state of the thermostat (environment) E of temperature *T*, namely,

(15) $$\begin{array}{c} \rho_{T}=\exp\left(-H_{E}/k_{B}T\right)/\mathrm{Tr}\left[\exp\left(-H_{E}/k_{B}T\right)\right], \end{array}$$

where:

(16) $$H_{E}=\sum_{i}\left[\frac{p_{i}^{2}}{2m_{i}}+\frac{1}{2}m_{i}\omega_{i}^{2}q_{i}^{2}\right]$$

is the Hamiltonian of the thermostat. The operator-valued random force F(t) is a family of non-commuting operators whose commutators are *c*-numbers. Its mean value is zero,

(17) $$\begin{array}{c} \langle F\left(t\right)\rangle \equiv \mathrm{Tr}\left[F\left(t\right)\rho_{T}\right]=0, \end{array}$$

and the symmetrized correlation function:

(18) $$C\left(t_{1},t_{2}\right)=\langle {\left[F\left(t_{1}\right);F\left(t_{2}\right)\right]}_{+}\rangle \equiv \frac{1}{2}\langle F\left(t_{1}\right)F\left(t_{2}\right)+F\left(t_{2}\right)F\left(t_{1}\right)\rangle$$

takes the form:

(19) $$\begin{array}{cc} C\left(t_{1},t_{2}\right)=C\left(t_{1}-t_{2}\right)= & \sum_{i}\frac{\hbar c_{i}^{2}}{2m_{i}\omega_{i}}\coth\left(\frac{\hbar \omega_{i}}{2k_{B}T}\right)\cos\left[\omega_{i}\left(t_{1}-t_{2}\right)\right]. \end{array}$$

The symmetrization is needed for $C(t_{1},t_{2})$ to be a real function with a correct limit in the classical case. We observe that it depends on the time difference $C\left(t_{1},t_{2}\right)=C\left(t_{1}-t_{2}\right)=C\left(\tau \right)$ for $\tau =t_{1}-t_{2}$. Statistical characteristics of the random force F(t) are similar to characteristics for a classical stationary Gaussian stochastic process, which models thermal equilibrium noise. Therefore, F(t) is called the Gaussian operator, which represents quantum thermal noise.

It is convenient to introduce the spectral function:

(20) $$\begin{array}{c} J\left(\omega \right)=\sum_{i}\frac{c_{i}^{2}}{m_{i}\omega_{i}^{2}}\delta \left(\omega -\omega_{i}\right). \end{array}$$

Then, the damping kernel Equation (13) can be expressed as:

(21) $$\gamma \left(\tau \right)=\int_{0}^{\infty }d\omega J\left(\omega \right)\cos\omega \tau$$

and the correlation function Equation (19) reads:

(22) $$C\left(\tau \right)=\int_{0}^{\infty }d\omega \;\frac{\hbar \omega }{2}\coth\left(\frac{\hbar \omega }{2k_{B}T}\right)J\left(\omega \right)\cos\omega \tau .$$

If we introduce the Fourier transforms of the dissipation and correlation functions,

(23) $$\gamma \left(\tau \right)=\int_{0}^{\infty }d\omega \;\hat{\gamma }\left(\omega \right)\cos\omega \tau ,\;C\left(\tau \right)=\int_{0}^{\infty }d\omega \;\hat{C}\left(\omega \right)\cos\omega \tau ,$$

then we see that the following equality:

(24) $$\hat{C}\left(\omega \right)=\frac{\hbar \omega }{2}\coth\left(\frac{\hbar \omega }{2k_{B}T}\right)\;\hat{\gamma }\left(\omega \right)$$

holds. We obtain the relation between the dissipation $\hat{\gamma }\left(\omega \right)$ and correlation $\hat{C}\left(\omega \right)$ spectra. This is what is named the fluctuation-dissipation theorem [12,13,14]. In this relation, quantum effects are incorporated via the prefactor in r.h.s. of Equation (24).

## 4. Dissipation Function

The influence of the thermostat E on the Brownian particle is manifested through the correlation function Equation (19) of the thermostat. If it is a finite quantum system, then its energy spectrum is discrete, and all its correlation functions are almost periodic in time. On the other hand, if the thermostat is an infinitely-extended system, then its correlation functions decay. Let us observe that if E is finite, then the spectral function J(ω) is a completely singular distribution (the sum in Equation (20) is over discrete values of *i*), and if E is infinite, then J(ω) is, at least on some intervals, a continuous function of ω (in the thermodynamic limit for E, the summation in Equation (20) is replaced by an integral over a frequency). Below and up to the end of the paper, we assume that the environment E is an infinite system.

In order to start an analysis of GLE Equation (12), one has to specify at least one of three quantities: the dissipation kernel γ(t), or the correlation function C(t) of the random force F(t), or the spectral function J(ω). We assume γ(t) to have the form:

(25) $$\gamma \left(t\right)=\frac{\gamma_{0}}{\tau_{c}}e^{-\left|t\right|/\tau_{c}}\cos\left(\Omega t\right)$$

with three non-negative parameters $\gamma_{0},\tau_{c}$ and Ω. The parameter $\gamma_{0}$ is the system-environment coupling strength, and $\tau_{c}$ defines decay or relaxation time and characterizes memory effects. Finally, Ω is the frequency in the relaxation process of the momentum (velocity). It is important to mention that $\tau_{c}$ is, via the fluctuation-dissipation theorem, the correlation time of quantum thermal fluctuations and appears in the correlation function $C(t_{1},t_{2})$ defined by Equation (18).

Why this form of the dissipation function? There are several reasons for this choice:

- In the classical limit, it is a direct relation between the dissipation kernel γ(t) and the correlation function C(t) : $C\left(t\right)=k_{B}T\gamma \left(t\right)$. Therefore, for Equation (25), the correlation function is sufficiently universal and has been considered for many systems.
- When Ω=0, it reduces to the exponential form and is known as a Drude model. Moreover, it has been considered in relation to colored noise problems.
- When Ω=0 and $\tau_{c}\to 0$, then γ(t) tends to the Dirac delta function, and the integral term reduces to $\gamma_{0}\dot{x}\left(t\right)$ (no memory effects), which is the well-known Stokes force with $\gamma_{0}$ interpreted as a friction (damping) coefficient. Here, the parameter $\gamma_{0}$ plays the role of coupling the strength of the Brownian particle to the thermostat.
- This form allows converting the integro-differential Equation (12) into a set of differential equations, which can be handled by known methods. It is important from a technical point of view to have a method of any sort to analyze Equation (12).

## 5. Generalized Langevin Equation as a Set of Differential Equations

The integral part of Equation (12) is the convolution of γ(t) and $\dot{x}\left(t\right)$. It suggests applying integral transforms like the Laplace or Fourier ones to solve it. Here, we exploit another method, which is based on the observation that if γ(t) fulfills a linear ordinary differential equation with constant coefficients, then Equation (12) can be converted to a set of ordinary differential equations. Note that the function γ(t) in Form (25) fulfills a differential equation of second order, which is similar to the Newton equation for a damped harmonic oscillator. We introduce auxiliary variables (in fact, operators) u(t) and v(t) by the relations:

(26) $$\begin{array}{cc} u(t) & =\mu \int_{0}^{t}e^{-\varepsilon (t-s)}\cos\left[\Omega \left(t-s\right)\right]\;p\left(s\right)ds, \end{array}$$

(27) $$\begin{array}{cc} v(t) & =\mu \int_{0}^{t}e^{-\varepsilon (t-s)}\sin\left[\Omega \left(t-s\right)\right]\;p\left(s\right)ds, \end{array}$$

(28) $$\begin{array}{cc} \mu & =\mu_{0}\;\varepsilon ,\;\mu_{0}=\frac{\gamma_{0}}{M},\;\varepsilon =\frac{1}{\tau_{c}}. \end{array}$$

Then, Equation (12) is converted to the following set of differential equations:

(29) $$\begin{array}{cc} \dot{x}\left(t\right) & =\frac{1}{M}p\left(t\right),\\ \dot{p}\left(t\right) & =-u\left(t\right)-U^{\prime }\left(x\left(t\right)\right)-\gamma \left(t\right)x\left(0\right)+F\left(t\right),\\ \dot{u}\left(t\right) & =\mu p(t)-\varepsilon u(t)-\Omega v(t),\\ \dot{v}\left(t\right) & =\Omega u(t)-\varepsilon v(t). \end{array}$$

To proceed further, we have to specify the form of the potential U(x). The simplest case is when U(x)=0, i.e., for the free particle. The second exactly solvable case is the harmonic oscillator with $U\left(x\right)=\left(1/2\right)M\omega_{0}^{2}x^{2}$. For other forms of U(x), the problem Equation (29) cannot be solved, and only mathematically uncontrolled approximations have been applied. The main elements of analysis of averaged kinetic energy are similar for both a free particle and a harmonic oscillator. However, a more pedagogical example is a free particle because the calculations are less tedious. It is the only reason for our choice U(x)=0. In such a case, in order to calculate the averaged kinetic energy, it is sufficient to consider the reduced set of equations:

(30) $$\begin{array}{cc} \dot{p}\left(t\right) & =-u(t)-\gamma (t)x(0)+F(t),\\ \dot{u}\left(t\right) & =\mu p(t)-\varepsilon u(t)-\Omega v(t),\\ \dot{v}\left(t\right) & =\Omega u(t)-\varepsilon v(t). \end{array}$$

It can be rewritten in the matrix form:

(31) $$\frac{d}{dt}X\left(t\right)=AX\left(t\right)+B\left(t\right),$$

where:

(32) $$\begin{array}{cc} X(t) & ={\left[p\left(t\right),u\left(t\right),v\left(t\right)\right]}^{T}, \end{array}$$

(33) $$\begin{array}{cc} B(t) & =\left(-\gamma (t)x(0)+F(t)\right){\left[\begin{array}{ccc} 1 & 0 & 0 \end{array}\right]}^{T} \end{array}$$

and T denotes the transpose of a matrix, which switches the row into the column. The matrix A has the form:

(34) $$A=\left[\begin{array}{ccc} 0 & -1 & 0\\ \mu & -\varepsilon & -\Omega \\ 0 & \Omega & -\varepsilon \end{array}\right].$$

The solution of the the non-homogeneous linear differential Equation (31) reads [6]:

(35) $$X\left(t\right)=R\left(t\right)X\left(0\right)+\int_{0}^{t}R\left(t-s\right)B\left(s\right)ds,\;R\left(t\right)=e^{At},$$

where:

(36) $$\begin{array}{cc} X(0) & ={\left[p\left(0\right),0,0\right]}^{T}. \end{array}$$

The spectrum of the matrix A and its invariant subspaces determine the time dependence of Equation (35). Now, the only problem is to determine the exponential of the matrix At, i.e., the matrix R(t), which can be computed in many ways. The authors of the paper [15] say about 19 ways. As they write: “In practice, consideration of computational stability and efficiency indicates that some of the methods are preferable to others, but that none are completely satisfactory”. The traditional way is to transform A into its Jordan canonical form. Here, we will use a less traditional method, namely the Putzer algorithm [16], in which the exponential of the matrix At can be computed knowing nothing more than the eigenvalues of the matrix A. Moreover, the algorithm does not require that the matrix A is diagonalizable. We think that this method is simple, elegant and suitable for presentation to students and younger researchers. It is described in Appendix A.

## 6. Average Kinetic Energy in Equilibrium

The operator of the kinetic energy $H_{k}\left(t\right)=p^{2}\left(t\right)/2M$ is expressed by the momentum p(t), which is the first component of the vector X(t) determined by Equation (35). We calculate its average in the long time limit t→∞ when a stationary state is approached. This stationary state is a thermal equilibrium state. The first component of X(t) is:

(37) $$p\left(t\right)=R_{11}\left(t\right)p\left(0\right)+\int_{0}^{t}R_{11}\left(t-s\right)\gamma \left(s\right)x\left(0\right)ds+\int_{0}^{t}R_{11}\left(t-s\right)F\left(s\right)ds,$$

where $R_{11}\left(t\right)$ is the first element of the matrix R(t). As is shown in Appendix A, elements of this matrix are exponentially decreasing functions of time. This means that the average value of the momentum 〈p(t)〉→0 as t→∞. To evaluate the average kinetic energy, we consider the symmetrized momentum-momentum correlation function $\langle {\left[p(t);p(u)\right]}_{+}\rangle$. In the long time limit, the first two terms of Equation (37) do not contribute to it, and only the last term contributes, yielding:

(38) $$\begin{array}{c} \langle {\left[p(t);p(s)\right]}_{+}\rangle =\int_{0}^{t}dt_{1}\int_{0}^{s}dt_{2}\;R_{11}\left(t-t_{1}\right)R_{11}\left(s-t_{2}\right)\langle {\left[F\left(t_{1}\right);F\left(t_{2}\right)\right]}_{+}\rangle . \end{array}$$

Now, we use the results of Section 3 and insert the expression for the correlation function Equation (23) of quantum thermal noise F(t) into Equation (38). The result is:

(39) $$\langle {\left[p(t);p(s)\right]}_{+}\rangle =\int_{0}^{\infty }d\omega \;\hat{C}\left(\omega \right)\int_{0}^{t}dt_{1}\int_{0}^{s}dt_{2}\;R_{11}\left(t-t_{1}\right)R_{11}\left(s-t_{2}\right)\cos\left[\omega \left(t_{1}-t_{2}\right)\right].$$

In particular, for t=s, it is the second statistical moment of the momentum,

(40) $$\langle p^{2}\left(t\right)\rangle =\int_{0}^{\infty }d\omega \;\hat{C}\left(\omega \right)\int_{0}^{t}dt_{1}\int_{0}^{t}dt_{2}\;R_{11}\left(t-t_{1}\right)R_{11}\left(t-t_{2}\right)\cos\left[\omega \left(t_{1}-t_{2}\right)\right].$$

We introduce new integration variables $\tau =t-t_{1}$ and $u=t-t_{2}$ to convert Equation (40) into the form:

(41) $$\langle p^{2}\left(t\right)\rangle =\int_{0}^{\infty }d\omega \;\hat{C}\left(\omega \right)\int_{0}^{t}d\tau \int_{0}^{t}du\;R_{11}\left(\tau \right)R_{11}\left(u\right)\cos\left[\omega \left(\tau -u\right)\right].$$

Finally, in the long time limit t→∞, average kinetic energy is obtained as:

(42) $$E_{k}=\lim_{t\to \infty }\frac{1}{2M}\langle p^{2}\left(t\right)\rangle =\frac{1}{2M}\int_{0}^{\infty }d\omega \;\hat{C}\left(\omega \right)I\left(\omega \right),$$

where:

(43) $$I\left(\omega \right)=\int_{0}^{\infty }d\tau \int_{0}^{\infty }du\;R_{11}\left(\tau \right)R_{11}\left(u\right)\cos\left[\omega \left(\tau -u\right)\right].$$

At this point, we use the fluctuation-dissipation relation Equation (24) to express the noise correlation spectrum $\hat{C}\left(\omega \right)$ by the noise dissipation spectrum $\hat{\gamma }\left(\omega \right)$, which, via Form (25) for the dissipation function γ(t) and its inverse Fourier transform, is given by:

(44) $$\begin{array}{c} \hat{\gamma }\left(\omega \right)=\frac{2}{\pi }\int_{0}^{+\infty }\gamma \left(t\right)\cos\left(\omega t\right)dt=\frac{\gamma_{0}\varepsilon^{2}}{\pi }\left[\frac{1}{{\left(\omega +\Omega \right)}^{2}+\varepsilon^{2}}+\frac{1}{{\left(\omega -\Omega \right)}^{2}+\varepsilon^{2}}\right]. \end{array}$$

The function I(ω) in Equation (43) is calculated from the relation Equation (A11) in Appendix A. Its explicit form is given by Equation (A16) in Appendix B. The numerator of I(ω) cancels with the denominator in Equation (44), and finally, we obtain:

(45) $$E_{k}=\int_{0}^{\infty }d\omega \;\frac{\hbar \omega }{4}\;\coth\left(\frac{\hbar \omega }{2k_{B}T}\right)\;P\left(\omega \right),$$

where:

(46) $$P\left(\omega \right)=\frac{2}{\pi }\frac{\mu_{0}\varepsilon^{2}\left(\omega^{2}+\varepsilon^{2}+\Omega^{2}\right)}{\omega^{6}+2\omega^{4}\left(\varepsilon^{2}-\Omega^{2}-\mu_{0}\varepsilon \right)+\omega^{2}\left(\mu_{0}^{2}\varepsilon^{2}+2\mu_{0}\varepsilon \Omega^{2}-2\mu_{0}\varepsilon^{3}+\Omega^{4}+2\Omega^{2}\varepsilon^{2}+\varepsilon^{4}\right)+\mu_{0}^{2}\varepsilon^{4}}.$$

We used the definition Equation (28) for $\mu =\mu_{0}\varepsilon$. We rewrite it in this form because both $\mu_{0}$ and ε have the unit of frequency (1/s). Moreover, $\mu_{0}=\gamma_{0}/M$ defines the rescaled coupling strength of the Brownian particle to the thermostat. The reciprocal $1/\mu_{0}=M/\gamma_{0}$ has the unit of time, and in the case of a classical free Brownian particle, it is the relaxation time of the particle velocity $v=\dot{x}$ (obtained from the Newton equation $M\dot{v}=-\gamma_{0}v$) or, equivalently, it is the correlation time occurring in the velocity-velocity correlation function.

The function P(ω) has no poles on a real axis because the denominator in Equation (46) can be rewritten in the form $x\left[{\left(x+c_{1}\right)}^{2}+c_{2}\right]+c_{3}>0$ for $x=\omega^{2},c_{1}=\varepsilon^{2}-\Omega^{2}-\mu_{0}\varepsilon$, positive $c_{2}=4\varepsilon^{2}\Omega^{2}$ and positive $c_{3}=\mu_{0}^{2}\varepsilon^{4}$. As a consequence, the integral in Equation (45) exists for any fixed values of parameters. Various forms of the expression Equation (45) have previously been derived [17,18,19]. However, the influence of the dissipation function Equation (25) on system properties, in particular in the context of average kinetic energy, has not been discussed.

The expression Equation (45) with the integrand Equation (46) is a quantum version of the equipartition theorem. It differs from its classical counterpart, like in Equation (4). The dependence of $E_{k}$ on temperature is involved in the integrand of Equation (45) and cannot be presented in an explicit, simple way. We note that there are four characteristic times $\tau_{1}=1/\mu_{0}=M/\gamma_{0},\tau_{2}=\tau_{c}=1/\varepsilon ,\tau_{3}=1/\Omega$ and $\tau_{4}=\hbar /k_{B}T$ (called the thermal time) and four corresponding frequencies $\mu_{0},\varepsilon ,\Omega$ and $k_{B}T/\hbar$ (called the Matsubara frequency), which all influence the temperature dependence of $E_{k}$.

Now, we want to take a look at the relation Equation (45) from another point of view. To this aim, we use the expression Equation (2) for the average kinetic energy of a single harmonic oscillator and rewrite Equation (45) in the form:

(47) $$E_{k}=\langle E_{k}\rangle =\int_{0}^{\infty }d\omega \;E_{k}\left(\omega \right)\;P\left(\omega \right).$$

The function P(ω) is positive, P(ω)>0, and normalizable (see Equation (A23) in Appendix C),

(48) $$\int_{0}^{\infty }d\omega \;P\left(\omega \right)=1.$$

Therefore, there exists a random variable ξ for which P is its probability distribution. From the mathematical point of view, Equation (47) is an average value of the function $E_{k}\left(\xi \right)$ of the random variable ξ (physicists frequently equate it with the integration variable). It allows presenting an interesting interpretation of the quantum equipartition theorem: the average kinetic energy $E_{k}$ of the Brownian particle is strongly related to the thermally-averaged kinetic energy $E_{k}\left(\omega \right)$ per one degree of freedom of oscillators of the environment. Because frequencies ω of oscillators are random variables, $E_{k}\left(\omega \right)$ has to be additionally averaged over all possible frequencies ω distributed according to the probability density P(ω) in which details of the particle-environment interaction are present via the parameters of the dissipation function γ(t).

## 7. Discussion

### 7.1. Average Kinetic Energy in Terms of Series

From the relation Equation (45), it is difficult to draw conclusions on the dependence of the average kinetic energy on the system parameters. Therefore, we present another form of $E_{k}$. To this aim, we exploit the series expansion [20]:

(49) $$x\coth\left(\frac{x}{2}\right)=2+4\sum_{n=1}^{\infty }\frac{x^{2}}{x^{2}+4\pi^{2}n^{2}}$$

which allows calculating the integral in Equation (45). More details are presented in Appendix C. From Equation (A25) in this appendix, we get the expression:

(50) $$\begin{array}{c} E_{k}=\frac{k_{B}T}{2}\left[1+2\sum_{n=1}^{\infty }\frac{\hbar \mu_{0}\;\hbar \varepsilon \left(\hbar \varepsilon +2\pi nk_{B}T\right)}{\hbar \mu_{0}\;\hbar \varepsilon \left(\hbar \varepsilon +2\pi nk_{B}T\right)+2\pi nk_{B}T{\left(\hbar \varepsilon +2\pi nk_{B}T\right)}^{2}+2\pi nk_{B}T{\left(\hbar \Omega \right)}^{2}}\right]. \end{array}$$

Here, the average kinetic energy is represented by an infinite series, and some information on $E_{k}$ can be inferred from this form. Since for n≥1, all terms under the sum are non-negative, hence $2E_{k}/k_{B}T$ is a lower bound for the energy $E_{k}$. Therefore, the kinetic energy in a quantum regime is always greater than the classical one. The term under the sum is a rational function of four characteristic energies $k_{B}T,\;\hbar \mu_{0},\;\hbar \varepsilon ,\;\hbar \Omega$. The numerator and denominator are the products of energy to a power of three, like, e.g., $\left(\hbar \mu_{0}\right)\;\left(\hbar \varepsilon \right)\;\left(k_{B}T\right)$. It is easy to observe that each term under the sum is a non-increasing function with respect to Ω because it occurs only in the denominator. Moreover, it can be shown that partial derivatives of each term with respect to $\mu_{0}$ and ε are non-negative, and it follows that all terms are non-decreasing with respect to $\mu_{0}$ and ε, respectively. As a consequence, $E_{k}$ is a non-increasing function of Ω and a non-decreasing function of $\mu_{0}$ and ε.

**Figure 1:**
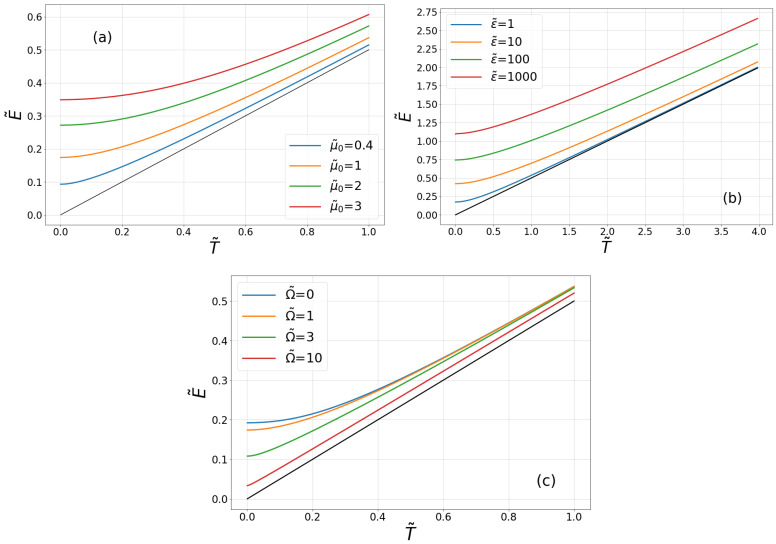
Average kinetic energy of the free Brownian particle as a function of rescaled temperature. (**a**) The influence of the rescaled particle-thermostat coupling strength ${\tilde{\mu }}_{0}=\mu_{0}/\varepsilon$. The rescaled energy is $\tilde{E}=E_{k}/\hbar \varepsilon$, and the rescaled temperature is $\tilde{T}=k_{B}T/\hbar \varepsilon$. The rescaled $\tilde{\Omega }=\Omega /\varepsilon =1$. (**b**) The influence of the rescaled inverse decay time $\tilde{\varepsilon }=\varepsilon /\mu_{0}$. The rescaled energy is $\tilde{E}=E_{k}/\hbar \mu_{0}$, and the rescaled temperature is $\tilde{T}=k_{B}T/\hbar \mu_{0}$. The rescaled $\tilde{\Omega }=\Omega /\mu_{0}=1$. (**c**) The influence of the rescaled frequency $\tilde{\Omega }=\Omega /\mu_{0}$. The rescaled energy is $\tilde{E}=E_{k}/\hbar \mu_{0}$, and the rescaled temperature is $\tilde{T}=k_{B}T/\hbar \mu_{0}$. The rescaled $\tilde{\varepsilon }=\varepsilon /\mu_{0}=1$.

At a fixed temperature, the kinetic energy $E_{k}$ is finite for all finite values of parameters and behaves in the following way (see Figure 1):

(i) $E_{k}$ increases monotonically to infinity when the coupling strength $\mu_{0}$ increases to infinity.
(ii) When the decay rate $\varepsilon =1/\tau_{c}$ grows from zero to infinity, $E_{k}$ grows from its classical value to infinity. In other words, for a very long decay time $\tau_{c}$, kinetic energy approaches its classical value Equation (4). When $\tau_{c}\to 0$, the kinetic energy diverges.
(iii) For Ω=0 (the Drude model), $E_{k}$ starts from some maximal value for a given set of parameters, and next, it decreases when Ω increases.

### 7.2. High Temperature Regime

We focus now on the regime of high temperatures. In this regime, when T→∞, we use the approximation:

(51) $$\coth\left(\frac{\hbar \omega }{2k_{B}T}\right)\approx \frac{2k_{B}T}{\hbar \omega }.$$

Then, Equation (45) reduces to the form:

(52) $$E_{k}=\frac{1}{2}k_{B}T$$

because the integral that occurs in Equation (45), according to Equation (48), is one. It is valid for any values of the parameters $\mu_{0},\varepsilon =1/\tau_{c}$ and Ω, which characterize and are involved in the dissipation kernel γ(t) defined in Equation (25). In particular, Equation (52) holds for weak, as well as strong system-environment interactions. The same Form (52) is obtained from Equation (50). Indeed, each term under the sum behaves as $T/T^{3}∼1/T^{2}$ when T→∞, and each term under the sum tends to zero. Only the first term survives, and Equation (50) is well approximated by Equation (52).

Let us now comment on the above approximations from the mathematical point of view. The approximation Equation (51) is valid when $\hbar \omega /2k_{B}T<<1$. However, let us observe that for any large value of $2k_{B}T$, there is a larger value of ℏω because the integration over ω in Equation (45) is to infinity and the inequality $\hbar \omega /2k_{B}T<<1$ is not satisfied for all ω. Nevertheless, Equation (52) is a correct approximation of Equation (45), and it can be shown by rigorous mathematical means. Moreover, it is supported by the method based on Equation (50). Because the series in Equation (50) is uniformly convergent, we can take the limit T→∞. Consequently, it implies Equation (52).

### 7.3. Low Temperature Regime

Let us note that the expression Equation (50) is not suitable for the calculation of the zero temperature limit because the indeterminate form ‘zero times infinity’ occurs. Equation (45) is more convenient. When temperature is low, T→0, the cotangent function can be approximated as follows:

(53) $$\coth\left(x\right)=1+2\;\frac{e^{-2x}}{1-e^{-2x}}\approx 1+2e^{-2x},\;x=\frac{\hbar \omega }{2k_{B}T}.$$

We insert this expression into Equation (45) and obtain:

(54) $$E_{k}=E_{0}+E_{1}\left(T\right),$$

where:

(55) $$E_{0}=\frac{1}{4}\int_{0}^{\infty }d\omega \;\hbar \omega \;P\left(\omega \right)$$

is the average kinetic energy for temperature T=0, i.e., when the thermostat is in a vacuum state and:

(56) $$E_{1}\left(T\right)=\frac{1}{2}\int_{0}^{\infty }d\omega \;\hbar \omega \;P\left(\omega \right)\;\exp\left[-\frac{\hbar \omega }{k_{B}T}\right]$$

is the first correction for small temperature T>0.

The kinetic energy $E_{0}$ at T=0 is finite for all finite values of parameters. Its parameters dependence is visualized in Figure 1, and it follows that:

(i) $E_{0}$ increases monotonically from zero to infinity when the coupling strength $\mu_{0}$ increases from zero to infinity.
(ii) When the decay rate $\varepsilon =1/\tau_{c}$ grows from zero to infinity, $E_{0}$ grows from zero to infinity.
(iii) For Ω=0 (the Drude model), $E_{0}$ starts from some maximal value for a given set of parameters, and next, it decreases when Ω increases.

### 7.4. Regime of Long Memory Time

The damping kernel γ(t) in the Langevin Equation (12) describes memory effects determined by the relaxation (decay) time $\tau_{c}$. For time scales shorter than $\tau_{c}$, memory effects can play an important role. For times longer than $\tau_{c}$, memory effects can be neglected, and the Ohmic model can be applied. Now, we consider the case of a long decay time $\tau_{c}$. More precisely, we assume that $\tau_{c}$ is much longer than the thermal Matsubara time $\hbar /k_{B}T$, namely,

(57) $$\begin{array}{c} \tau_{c}=\frac{1}{\varepsilon }>>\frac{\hbar }{2\pi k_{B}T}. \end{array}$$

In other words, $\hbar \varepsilon <<2\pi k_{B}T$, and then, $\hbar \varepsilon +2\pi nk_{B}T\approx 2\pi nk_{B}T$ in Equation (50). In this regime, Equation (50) takes the form:

(58) $$\begin{array}{c} E_{k}=\frac{k_{B}T}{2}\left[1+2\sum_{n=1}^{\infty }\frac{\hbar^{2}\mu_{0}\;\varepsilon }{\hbar^{2}\left(\mu_{0}\varepsilon +\Omega^{2}\right)+{\left(2\pi nk_{B}T\right)}^{2}}\right]. \end{array}$$

We can use Formula (49) to rewrite the above equation in a more compact form as:

(59) $$\begin{array}{c} E_{k}=\frac{k_{B}T}{2}\left[\frac{\Omega^{2}}{\varepsilon \mu_{0}+\Omega^{2}}+\frac{\hbar \varepsilon \mu_{0}}{2k_{B}T\sqrt{\varepsilon \mu_{0}+\Omega^{2}}}\coth\left(\frac{\hbar \sqrt{\varepsilon \mu_{0}+\Omega^{2}}}{2k_{B}T}\right)\right]. \end{array}$$

For the Drude model, when Ω=0, it reduces to the following equation:

(60) $$\begin{array}{c} E_{k}=\frac{1}{4}\hbar \sqrt{\varepsilon \mu_{0}}\coth\left(\frac{\hbar \sqrt{\varepsilon \mu_{0}}}{2k_{B}T}\right). \end{array}$$

This is a surprising result because it looks like Equation (2) for the averaged kinetic energy of the oscillator with its redefined eigenfrequency $\omega_{0}=\sqrt{\varepsilon \mu_{0}}$. Remember that the relation Equation (57) should be satisfied, and this means that:

(61) $$\begin{array}{c} \tau_{c}>>1.21\times 10^{-12}\frac{1}{T}\;sK. \end{array}$$

e.g., for a temperature of 1 Kelvin, $\tau_{c}>>10^{-12}$ s, while for $10^{-4}$ Kelvin, $\tau_{c}>>10^{-8}$ s. Therefore, for higher temperatures, it is easier to fulfil this condition.

## 8. Conclusions

In summary, in the framework of the Generalized Langevin Equation (GLE), we studied the kinetic energy of a quantum Brownian particle in an equilibrium state. We assumed a relatively general form of the integral kernel of GLE and analyzed kinetic energy in selected regimes like high or low temperature limits. In the high temperature limit, the standard result of the classical statistical mechanics of the equipartition energy $E_{k}=k_{B}T/2$ is valid independently of the strength of the system-environment interaction, decay of the dissipation kernel and its frequency parameter. In the zero temperature regime, when fluctuations of the environment vacuum influence the system, average kinetic energy of the free Brownian particle is non-zero, and its value depends on parameters of the dissipation function. In particular, it is an increasing function of the coupling strength quantified by the parameter $\gamma_{0}$ or the rescaled parameter $\mu_{0}$. It is also interesting that when the decay time $\tau_{c}$ in the dissipation kernel tends to zero, average kinetic energy grows to infinity [19]. This means that the assumption of zero decay time is not physically correct, and memory effects have to be taken into account.

We propose a new interpretation of the relation Equation (45): the average kinetic equation of the Brownian particle equals the thermally-averaged kinetic energy per one degree of freedom of thermostat oscillators, additionally averaged over randomly-distributed oscillator frequencies. Our conjecture is that this interpretation is not only for this particular system, but it may be universal, at least for some classes of systems.

We considered an exactly solved model of a quantum open system. In a general case, only approximate results can be derived, e.g., in the so-called quantum Smoluchowski regime, an effective evolution equation of the Fokker–Planck type has been derived [21,22] and applied to many problems such as quantum diffusion [23,24] or quantum Brownian motors [25]. Some extensions to include reservoirs consisting of non-linear oscillators have been proposed [26]. Finally, it is worth mentioning a novel and alternative approach to attack the problem of a quantum Brownian particle, which is based on an adjoint master equation for a generic operator of the system [27].

## Appendix A. Putzer Algorithm

In the relation Equation (35), the exponential of the matrix At is needed. We calculate it applying the Putzer method [16]. The exponential can be presented in the form:

(A1) $$e^{At}=R\left(t\right)=r_{1}\left(t\right)I+r_{2}\left(t\right)P_{1}+r_{3}\left(t\right)P_{2},$$

where *I* is the identity matrix; the matrices $P_{1}$ and $P_{2}$ are determined by the relations:

(A2) $$P_{1}=A-\lambda_{1}I,\;P_{2}=\left(A-\lambda_{2}I\right)P_{1}$$

and $\lambda_{j}\;\left(j=1,2,3\right)$ are the eigenvalues (in any order and not necessarily distinct) of the matrix A. The functions $r_{j}\left(t\right)$ takes the form:

(A3) $$\begin{array}{ccc} r_{1}\left(t\right) & = & e^{\lambda_{1}t}, \end{array}$$

(A4) $$\begin{array}{ccc} r_{2}\left(t\right) & = & e^{\lambda_{2}t}\int_{0}^{t}e^{-\lambda_{2}u}\;r_{1}\left(u\right)\;du=\frac{e^{\lambda_{1}t}-e^{\lambda_{2}t}}{\lambda_{1}-\lambda_{2}}, \end{array}$$

(A5) $$\begin{array}{ccc} r_{3}\left(t\right) & = & e^{\lambda_{3}t}\int_{0}^{t}e^{-\lambda_{3}u}\;r_{2}\left(u\right)\;du=\frac{1}{\lambda_{1}-\lambda_{2}}\left[\frac{e^{\lambda_{1}t}-e^{\lambda_{3}t}}{\lambda_{1}-\lambda_{3}}-\frac{e^{\lambda_{2}t}-e^{\lambda_{3}t}}{\lambda_{2}-\lambda_{3}}\right]. \end{array}$$

The eigenvalues $\lambda_{j}$ are the roots of the characteristic polynomial |A−λI|=0, which explicitly reads:

(A6) $$\lambda^{3}+2\varepsilon \lambda^{2}+\left(\mu +\varepsilon^{2}+\Omega^{2}\right)\lambda +\varepsilon \mu =0.$$

All eigenvalues have negative real parts, i.e., Reλj<0. The necessary and sufficient conditions for this to hold are the Routh–Hurwitz conditions, which for the cubic equation:

(A7) $$\lambda^{3}+a_{2}\lambda^{2}+a_{1}\lambda +a_{0}=0$$

take the form:

(A8) $$a_{2}=2\varepsilon >0,\;a_{0}=\varepsilon \mu >0,\;a_{2}a_{1}-a_{0}=2\varepsilon \left(\varepsilon^{2}+\Omega^{2}\right)+\varepsilon \mu >0.$$

As a consequence, all functions $r_{j}\left(t\right)$ are decreasing functions of time.

The explicit forms of the matrices in Equation (A1) read:

(A9) $$\begin{array}{cc} P_{1} & =-\left[\begin{array}{ccc} \lambda_{1} & 1 & 0\\ -\mu \;\; & \lambda_{1}+\varepsilon \; & \Omega \;\\ 0\; & -\Omega \; & \lambda_{1}+\varepsilon \; \end{array}\right], \end{array}$$

(A10) $$\begin{array}{cc} P_{2} & =\left[\begin{array}{ccc} \lambda_{1}\lambda_{2}-\mu & \lambda_{1}+\lambda_{2}+\varepsilon & \Omega \\ -\mu \left(\lambda_{1}+\lambda_{2}+\varepsilon \right)\;\; & \left(\lambda_{1}+\varepsilon \right)\left(\lambda_{2}+\varepsilon \right)-\mu -\Omega^{2} & \Omega \left(\lambda_{1}+\lambda_{2}+2\varepsilon \right)\\ \mu \Omega & -\Omega \left(\lambda_{1}+\lambda_{2}+2\varepsilon \right) & \left(\lambda_{1}+\varepsilon \right)\left(\lambda_{2}+\varepsilon \right)-\Omega^{2} \end{array}\right]. \end{array}$$

What we need in Equation (43) is the first element $R_{11}\left(t\right)$ of the matrix R(t). We exploit the above Formulas (A1)–(A10) and get:

(A11) $$R_{11}\left(t\right)=\left(\lambda_{2}\lambda_{3}-\mu \right)b_{1}e^{\lambda_{1}t}-\left(\lambda_{1}\lambda_{3}-\mu \right)b_{2}e^{\lambda_{2}t}+\left(\lambda_{1}\lambda_{2}-\mu \right)b_{3}e^{\lambda_{3}t},$$

where:

(A12) $$\begin{array}{cc} b_{1} & =\frac{1}{{\lambda_{1}}^{2}-\lambda_{1}\lambda_{2}-\left(\lambda_{1}-\lambda_{2}\right)\lambda_{3}}, \end{array}$$

(A13) $$\begin{array}{cc} b_{2} & =-\frac{1}{\lambda_{1}\lambda_{2}-{\lambda_{2}}^{2}-\left(\lambda_{1}-\lambda_{2}\right)\lambda_{3}}, \end{array}$$

(A14) $$\begin{array}{cc} b_{3} & =\frac{1}{\lambda_{1}\lambda_{2}-\left(\lambda_{1}+\lambda_{2}\right)\lambda_{3}+{\lambda_{3}}^{2}}. \end{array}$$

## Appendix B. Calculation of *I*(ω)

In this appendix, we calculate the function I(ω) defined by Equation (43). The integrand $R_{11}\left(t\right)$ is the exponential function Equation (A11), and the double integral in Equation (43) can easily be calculated. The result reads:

(A15) $$\begin{array}{cc} I(\omega ) & =\frac{{\left(\lambda_{2}\lambda_{3}-\mu \right)}^{2}b_{1}^{2}}{{\lambda_{1}}^{2}+\omega^{2}}+\frac{{\left(\lambda_{1}\lambda_{3}-\mu \right)}^{2}b_{2}^{2}}{{\lambda_{2}}^{2}+\omega^{2}}+\frac{{\left(\lambda_{1}\lambda_{2}-\mu \right)}^{2}b_{3}^{2}}{{\lambda_{3}}^{2}+\omega^{2}}\\ & -\frac{2\;\left(\lambda_{1}\lambda_{2}+\omega^{2}\right)\left(\lambda_{1}\lambda_{3}-\mu \right)\left(\lambda_{2}\lambda_{3}-\mu \right)b_{1}b_{2}}{{\lambda_{1}}^{2}{\lambda_{2}}^{2}+\omega^{4}+\left({\lambda_{1}}^{2}+{\lambda_{2}}^{2}\right)\omega^{2}}\\ & +\frac{2\;\left(\lambda_{1}\lambda_{2}-\mu \right)\left(\lambda_{1}\lambda_{3}+\omega^{2}\right)\left(\lambda_{2}\lambda_{3}-\mu \right)b_{1}b_{3}}{{\lambda_{1}}^{2}{\lambda_{3}}^{2}+\omega^{4}+\left({\lambda_{1}}^{2}+{\lambda_{3}}^{2}\right)\omega^{2}}\\ & -\frac{2\;\left(\lambda_{1}\lambda_{2}-\mu \right)\left(\lambda_{1}\lambda_{3}-\mu \right)\left(\lambda_{2}\lambda_{3}+\omega^{2}\right)b_{2}b_{3}}{{\lambda_{2}}^{2}{\lambda_{3}}^{2}+\omega^{4}+\left({\lambda_{2}}^{2}+{\lambda_{3}}^{2}\right)\omega^{2}} \end{array}$$

Inserting the coefficients $b_{j}\;\left(j=1,2,3\right)$ from Equations (A12)–(A14) and using Vieta’s formulas for the roots of the polynomial Equation (A6), we obtain the final expression:

(A16) $$I\left(\omega \right)=\frac{\left[{\left(\omega +\Omega \right)}^{2}+\varepsilon^{2}\right]\left[{\left(\omega -\Omega \right)}^{2}+\varepsilon^{2}\right]}{\omega^{6}+2\omega^{4}\left(\varepsilon^{2}-\Omega^{2}-\mu \right)+\omega^{2}\left(\mu^{2}+2\mu \Omega^{2}-2\mu \varepsilon^{2}+\Omega^{4}+2\Omega^{2}\varepsilon^{2}+\varepsilon^{4}\right)+\mu^{2}\varepsilon^{2}}$$

This function occurs in Equation (42) for the average kinetic energy.

## Appendix C. Series Expansion for Kinetic Energy

We will present Equation (45) in the form of a series. To this aim, we introduce the dimensionless integral variable θ and parameters,

(A17) $$\begin{array}{c} \theta =\tau_{T}\omega ,\;\tau_{T}=\frac{\hbar }{k_{B}T} \end{array}$$

(A18) $$\begin{array}{c} {\hat{\mu }}_{0}=\tau_{T}\mu_{0},\;\hat{\varepsilon }=\tau_{T}\varepsilon ,\;\hat{\Omega }=\tau_{T}\Omega \end{array}$$

Next, we exploit the series expansion [20]:

(A19) $$\theta \coth\left(\frac{\theta }{2}\right)=2+4\sum_{n=1}^{\infty }\frac{\theta^{2}}{\theta^{2}+4\pi^{2}n^{2}}$$

Using the above expansion, we obtain the integrand of Equation (45) in the form of uniformly-convergent series. Therefore, we can utilize the Weierstrass theorem and integrate term by term as follows:

(A20) $$\begin{array}{cc} \frac{2E_{k}}{kT} & =I_{0}+\sum_{n=1}^{\infty }I_{n} \end{array}$$

(A21) $$\begin{array}{cc} I_{0} & =\frac{2}{\pi }\int_{0}^{\infty }\frac{{\hat{\mu }}_{0}{\hat{\varepsilon }}^{2}\left(\theta^{2}+{\hat{\Omega }}^{2}+{\hat{\varepsilon }}^{2}\right)}{\theta^{6}-2\theta^{4}\left({\hat{\Omega }}^{2}+{\hat{\mu }}_{0}\hat{\varepsilon }-{\hat{\varepsilon }}^{2}\right)+\theta^{2}\left({\hat{\Omega }}^{4}+2{\hat{\Omega }}^{2}{\hat{\mu }}_{0}\hat{\varepsilon }+2{\hat{\Omega }}^{2}{\hat{\varepsilon }}^{2}+{\hat{\mu }}_{0}^{2}{\hat{\varepsilon }}^{2}-2{\hat{\mu }}_{0}{\hat{\varepsilon }}^{3}+{\hat{\varepsilon }}^{4}\right)+{\hat{\mu }}_{0}^{2}{\hat{\varepsilon }}^{4}}d\theta \end{array}$$

(A22) $$\begin{array}{cc} I_{n} & =\frac{1}{\pi }\int_{0}^{\infty }\frac{{\hat{\mu }}_{0}{\hat{\varepsilon }}^{2}\left(\theta^{2}+{\hat{\Omega }}^{2}+{\hat{\varepsilon }}^{2}\right)}{\theta^{6}-2\theta^{4}\left({\hat{\Omega }}^{2}+{\hat{\mu }}_{0}\hat{\varepsilon }-{\hat{\varepsilon }}^{2}\right)+\theta^{2}\left({\hat{\Omega }}^{4}+2{\hat{\Omega }}^{2}{\hat{\mu }}_{0}\hat{\varepsilon }+2{\hat{\Omega }}^{2}{\hat{\varepsilon }}^{2}+{\hat{\mu }}_{0}^{2}{\hat{\varepsilon }}^{2}-2{\hat{\mu }}_{0}{\hat{\varepsilon }}^{3}+{\hat{\varepsilon }}^{4}\right)+{\hat{\mu }}_{0}^{2}{\hat{\varepsilon }}^{4}}\frac{4\theta^{2}}{4\pi^{2}k^{2}+\theta^{2}}d\theta \end{array}$$

For all cases, the integrands are rational functions. The polynomial degrees of denominators are higher by four than the degree of polynomials in the numerators, and therefore, all integrals exist. We can use an elegant method of the residue theorem to calculate the integrals [6]. Manual calculations are long and tedious, but nowadays, this problem can be overcome by using any computer algebra system. We used SymPy (the Python library) for symbolic mathematics.

The integrand of the zero-th term $I_{0}$ is the same as Equation (46). The denominator of the integrand has six roots on the complex plane with three poles in the upper half-plane and three poles in the lower half-plane. We can choose a contour closed in the upper half-plane, and from the residue method, we obtain:

(A23) $$I_{0}=1\;\Rightarrow \int_{0}^{\infty }d\omega \;P\left(\omega \right)=1.$$

For remaining terms, we obtain the expression for $I_{n}$ in the following form:

(A24) $$I_{n}=\frac{2{\hat{\mu }}_{0}\hat{\varepsilon }\left(\hat{\varepsilon }+2\pi n\right)}{{\hat{\mu }}_{0}\hat{\varepsilon }\left(\hat{\varepsilon }+2\pi n\right)+2\pi n{\left(\hat{\varepsilon }+2\pi n\right)}^{2}+2\pi {\hat{\Omega }}^{2}n}.$$

Finally,

(A25) $$\begin{array}{c} \frac{2E_{k}}{k_{B}T}=1+\sum_{n=1}^{\infty }\frac{2{\hat{\mu }}_{0}\hat{\varepsilon }\left(\hat{\varepsilon }+2\pi n\right)}{{\hat{\mu }}_{0}\hat{\varepsilon }\left(\hat{\varepsilon }+2\pi n\right)+2\pi n{\left(\hat{\varepsilon }+2\pi n\right)}^{2}+2\pi {\hat{\Omega }}^{2}n}. \end{array}$$

Using this method, we obtain a form that is more convenient for discussing the general properties of kinetic energy with respect to system parameters. We presented this in Section 7.
